# Welcome to *Carbon Balance and Management*

**DOI:** 10.1186/1750-0680-1-1

**Published:** 2006-06-27

**Authors:** Robert Dickinson

**Affiliations:** 1 Georgia Institute of Technology, Atlanta, USA

## Abstract

We are pleased to announce the launch of *Carbon Balance and Management*, a new online open access journal published by BioMed Central.

## Carbon Balance and Management

*Carbon Balance and Management *is a new open access, peer-reviewed online journal that encompasses all aspects of research aimed at developing a comprehensive, policy-relevant understanding of the global carbon cycle [1].

Advancement in the union of the two issues indicted by the journal's title will be a very important element of future global economic and societal development. We must develop predictive and observational capabilities to determine how carbon systems are changing now and how they will be changing in the future, and we must develop management tools and wisdom on the related human systems in order to better determine the uses and environmental storage of carbon; this must all be done consistent with maintaining the integrity and values of our global environment.

We expect many of the papers submitted to *Carbon Balance and Management *to be at the forefront of research on how these multiple objectives can be better met. The journal's open access policy [2,3] combined with its fast publishing system, should facilitate the rapid communication that is needed to address these issues.

*Carbon Balance and Management *considers several types of article: research, methodology, review, and commentary. Manuscripts submitted through the online submission system are assigned to a Handling Editor, a member of the editorial board [4], who will send the manuscript to three anonymous reviewers and use their advice to reach a fast editorial decision.

The traditional business model for scientific publishers relies on restricting access to published research, in order to recoup the costs of the publication process. This restriction of access to published research prevents full use being made of digital technologies.

The financial vein of the open access model is the Article Processing Charge (APC). Article-processing charges cover the cost of the publication process to allow free and immediate access to the research articles. Due to the low expense of electronic publication, these costs are no more than the page charges for many subscription journals.

Authors publishing in *Carbon Balance and Management *retain the copyright to their work, licensing it under the Creative Commons Attribution License [5]. This agreement ensures that articles are freely accessible upon publication. In addition, authors who submit from an organization with BioMed Central membership will be able to publish for free or at a discounted rate [6].

The Editors of *Carbon Balance and Management *look forward to receiving your submissions.
